# Pathological and immunological features of autoinflammatory syndrome associated with lymphedema (AISLE)

**DOI:** 10.1186/1546-0096-13-S1-O25

**Published:** 2015-09-28

**Authors:** A Gul, H Ozdogan, S Ugurlu, O Kasapcopur, N Buyukbabani, U Emekli, Z Emrence, D Ustek

**Affiliations:** 1 Istanbul University, Istanbul Faculty of Medicine, Department of Internal Medicine, Istanbul, Turkey; 2 Istanbul University, Cerrahpasa Faculty of Medicine, Department of Internal Medicine, Division of Rheumatology, Istanbul, Turkey; 3 Istanbul University, Cerrahpasa Faculty of Medicine, Department of Paediatrics, Istanbul, Turkey; 4 Istanbul University, Istanbul Faculty of Medicine, Department of Pathology, Istanbul, Turkey; 5 Istanbul University, Istanbul Faculty of Medicine, Department of Plastic Surgery, Istanbul, Turkey; 6 Istanbul University, Institute for Experimental Medicine, Department of Genetics, Istanbul, Turkey

## Background

We recently described a new autoinflammatory syndrome associated with lymphedema (AISLE), and a frameshift mutation in the MDFIC gene was identified by homozygosity mapping and targeted sequencing using DNA samples of a consanguineous Turkish family as the cause of this syndrome. The same mutation in the MDFIC gene was identified in an Italian patient with similar clinical findings. Clinical findings of these patients are characterized by recurrent attacks of fever, erythematous/urticarial rash with hyperesthesia, myalgia, serositis, chylous serosal effusions, edema/lymphedema on the face and extremities. These patients gradually developed a permanent lymphedema in the same areas affected during attacks. On the other hand, there is limited information about the functions of MDFIC gene, which include transcriptional regulator interacting with cellular transcription factors, including such as axin and wnt/b-catenin signaling pathway. Although, its expression has been documented in immune cells, no clear function has been described in immune mediated or inflammatory conditions.

## Methods

We evaluated the clinical course and laboratory investigations of the patients. We investigated the gene expression patterns in peripheral blood mononuclear cells in one of the patients and his parents following LPS stimulation to understand involved pathways in MDFIC-related inflammatory changes using an Illumina gene expression kit. We also evaluated pathological specimens obtained from the same patient during surgical resection of the giant scrotal tissue affected from lymphedema. Detailed immunohistochemical analyses were done to define the characteristics of lymphatics in involved tissues.

## Results

LPS stimulation resulted in a significant deviation in gene expression pattern in the AISLE patient compared to the expression patterns of one of his parents and un-related healthy controls. Expression of CCL8, IFITM3, IFIT1, IFIT3, IFIT2, ISG15, OAS3 genes showed more than two times decrease following LPS stimulation, and these gene expression changes were interpreted as a negative interferon signature. Pathological examinations revealed lower than expected number of lymphatic vessels as well as rare enlarged lymhatics in scrotal tissues with lymphedema. Regarding two of the Turkish patients’ treatments, no clear response was observed to monoclonal anti-IL-1 beta canakinumab antibody. However, both patients are doing well with daily anakinra injections. The first patient experienced no adverse event during and following two operations for giant scrotal lymphedema, and a normal recovery period was observed.

## Conclusions

Gene expression findings indicating a negative interferon response following LPS stimulation need to be confirmed in other two patients and cell line models. Current immunological and pathological findings suggest an interferon-signature mediated inflammatory response defect, which may affect lymphatic endothelial cells and their survival, leading to gradual decrease in the number of lymphatic vessels and development of lymphedema. Also differential response to anakinra but not canakinumab may suggest possible contribution of IL-1 alpha to the pathogenesis of AISLE.

